# Purple Corn Extract as Anti-allodynic Treatment for Trigeminal Pain: Role of Microglia

**DOI:** 10.3389/fncel.2018.00378

**Published:** 2018-11-05

**Authors:** Giulia Magni, Alessandra Marinelli, Daniele Riccio, Davide Lecca, Chiara Tonelli, Maria P. Abbracchio, Katia Petroni, Stefania Ceruti

**Affiliations:** ^1^Department of Pharmacological and Biomolecular Sciences, Università degli Studi di Milano, Milan, Italy; ^2^Department of Biosciences, Università degli Studi di Milano, Milan, Italy

**Keywords:** microglia, anthocyanins, inflammatory trigeminal pain, allodynia, functional foods

## Abstract

Natural products have attracted interest in the search for new and effective analgesics and coadjuvant approaches to several types of pain. It is in fact well known that many of their active ingredients, such as anthocyanins (ACNs) and polyphenols, can exert potent anti-inflammatory actions. Nevertheless, their potential beneficial effects in orofacial painful syndromes have not been assessed yet. Here, we have evaluated the preventive effect of an ACN-enriched purple corn extract against the development of orofacial allodynia, in comparison with isogenic yellow corn extract containing only polyphenols. Orofacial allodynia developed following induction of temporomandibular joint (TMJ) inflammation in male rats, due to the injection of Complete Freund’s Adjuvant (CFA), and was evaluated by von Frey filaments. Animals drank purple or yellow corn extracts or water starting from 11 days before induction of inflammation and up to the end of the experiment 3 days later. To highlight possible additive and/or synergic actions, some animals also received the anti-inflammatory drug acetyl salicylic acid (ASA). In parallel with the evaluation of allodynia, we have focused our attention on the activation of microglia cells in the central nervous system (CNS), as it is well-known that they significantly contribute to neuronal sensitization and pain. Our data demonstrate that purple corn extract is as effective as ASA in preventing the development of orofacial allodynia, and only partial additive effect is observed when the two agents are co-administered. Yellow corn exerted no effect. Multiple mechanisms are possibly involved in the action of purple corn, including reduction of trigeminal macrophage infiltration and the shift of microglia cell polarization to an anti-inflammatory phenotype. In fact, in rats receiving yellow corn or water microglia cells show thick, short cell processes typical of activated cells. Conversely, thinner and longer microglia cell processes are observed in the brainstem of animals drinking purple corn extract; shape changes are accompanied by a reduction in the expression of pro-inflammatory molecules and increased production of anti-inflammatory mediators. Administration of purple corn extracts therefore represents a possible low-cost and easy way to reduce trigeminal-associated pain in various pathological conditions also thanks to the modulation of microglia reactivity.

## Introduction

Alternative and complementary approaches to currently available painkillers are now attracting researchers and clinicians, with the dual aim of reducing drug-associated side effects and to possibly find more effective treatments for conditions lacking satisfactory management protocols. This is the case of various types of trigeminal-related pain, including temporomandibular joint (TMJ) pain which arises from a heterogeneous group of musculoskeletal and neuromuscular problems (Gauer and Semidey, 2015). TMJ pain is characterized by a strong inflammatory component, and the first line pharmacological treatment is currently represented by non-steroidal anti-inflammatory drugs (NSAIDs), which sometimes lead to only partial pain relief accompanied by the risk of the well-known and potentially life-threatening side effects (i.e., bleeding, gastrointestinal ulcerations, drug/drug interactions in the case of multitherapy, allergic reactions in a percentage of subjects). Benzodiazepines and capsaicin are also utilized (Häggman-Henrikson et al., 2017).

The lack of effective therapies is also based on the common idea that analgesics should target neurons. In recent years, research has instead demonstrated a significant contribution to pain of non-neuronal cells, both in the central nervous system (CNS) and in periphery. We demonstrated that brainstem microglial cells and trigeminal ganglion glial cells are activated by the induction of TMJ pain in adult male rats (Villa et al., 2010; Magni et al., 2015), thus contributing to the development of a pro-inflammatory milieu which in turn is responsible for the development of neuronal sensitization and the chronicization of pain.

Naturally-occurring anti-inflammatory molecules, such as anthocyanins (ACNs) and polyphenols which can be easily administered to patients as extracts or functional foods, have attracted the interest of the scientific community as adjuvants and complements to drug therapy. In particular, evidence that consumption of ACN-rich food promotes health is supported by epidemiological and preclinical studies performed with different dietary sources of ACNs, showing that they can protect against cancer, cardiovascular disease and age-related neurodegenerative diseases, all characterized by the development of inflammation (Joseph et al., 1999; Butelli et al., 2008; Toufektsian et al., 2008; He and Giusti, 2010; Tsuda, 2012; Cassidy et al., 2013; Jennings et al., 2014). Furthermore, dietary ACNs from purple corn have been shown to protect against myocardial damages induced by Doxorubicin, an anthracycline widely used as chemotherapeutic drug against a variety of cancer types (Petroni et al., 2017). Interestingly, purple corn extract does not affect the cytotoxic effects of Doxorubicin on tumor cells, suggesting it as a beneficial functional food for patients subjected to anthracycline chemotherapy (Petroni et al., 2017).

Based on these premises, we have deemed it interesting to evaluate the possible anti-allodynic action of the administration of a purple corn extract (Red extract) to rats exposed to inflammatory TMJ pain, in parallel with isogenic yellow corn supplement (Yellow extract) as control, alone or in combination with acetyl salicylic acid (ASA) as reference drug. Results show a significant amelioration of allodynia in rats drinking Red extract, which is fully comparable to ASA-induced effect. The positive effect on painful behavior is linked to both peripheral and central actions and involves the normalization of glial cell activation.

## Materials and Methods

### Animals

Male adult Sprague-Dawley rats (200–250 g; Charles River Lab., Calco, Italy) were utilized, as previously described (Magni et al., 2015). Animals were housed under standard conditions (temperature 22 ± 2°C; relative humidity 50 ± 10%; artificial light 12 h light/dark cycle with lights on at 7 AM). Water and food were provided *ad libitum* until the beginning of corn supplement administration (see below). Experiments were performed between 9:00 and 16:00 in the rat housing room, whereas sacrifice took place in a separate operatory room. The study was carried out in accordance with the principles of the Basel Declaration and recommendations of the Committee for Research and Ethical Issues of the International Association for the Study of Pain (IASP), and with National and European regulations regarding the protection of animals used for experimental and other scientific purposes (D.L. 26_2014; 2010/63/UE), as well as following the Society for Neuroscience’s policies on the Use of Animals and Humans in Neuroscience Research. The study protocols have been approved by the Council of the Department of Pharmacological and Biomolecular Sciences and by the ethics committee (OPBA) of the Università degli Studi di Milano (Milan, Italy). Formal authorization (#736/2015-PR) was granted by the Italian Ministry of Health, after positive evaluation by the Italian Institute of Health, Rome. All efforts have been made to minimize animal suffering, and to reduce the number of animals to a minimum.

### Preparation of Corn Supplements and Their Administration to Animals

Red extract was produced by SVEBA Srl (Appiano Gentile, Italy) from *B*1 P*l1* purple corn cobs through extraction with ethanol: H_2_O (30:70 v/v) at 55°C for 1 h, titrated to a concentration of 4% ACNs and spray-dried to a final concentration of 31.25 mg/g of ACNs. Yellow extract was produced from *b*1 p*l1* yellow corn cobs using identical protocols and volumes as Red extract. A comparable total amount of flavonoids in Red and Yellow extract, apart from ACNs, as in the original raw plant material was previously verified (Petroni et al., 2017).

**Figure 1:**
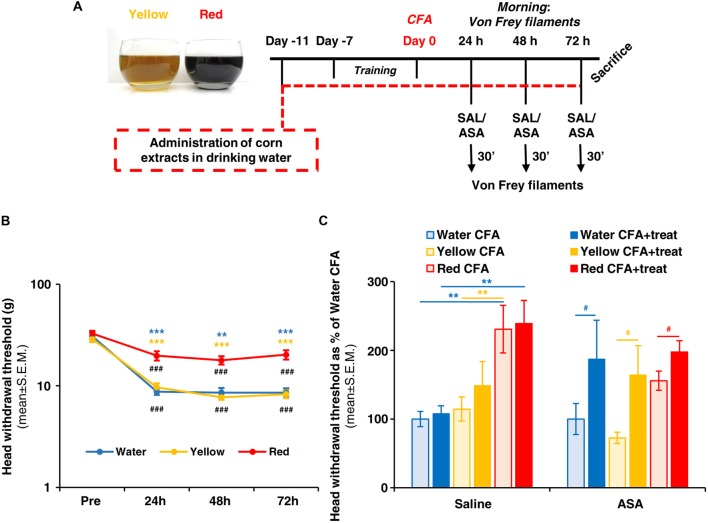
Red extract is as effective as acetyl salicylic acid (ASA) in reverting trigeminal allodynia. **(A)** Schematic representation of the experimental protocol utilized in this study. See “Materials and Methods” section for details. **(B)** Evaluation of facial allodynia in rats drinking water, Yellow or Red extracts and exposed to trigeminal sensitization by Complete Freund’s Adjuvant (CFA) injection in the temporomandibular joint (TMJ). The head withdrawal threshold was evaluated at 24, 48 and 72 h post CFA injection in the morning (see **(A)**), in 25 rats for the Water group and 33 rats each for the Yellow and Red groups. ^###^*p* < 0.001 with respect to corresponding “PRE” values, one-way ANOVA followed by Tukey’s test; **p < 0.01 ***p < 0.001 with respect to purple corn supplement, one-way ANOVA followed by Bonferroni test. **(C)** Evaluation of facial allodynia in CFA-injected animals drinking water, Yellow or Red extracts and treated with ASA or saline. For each condition (i.e., rats drinking water, yellow or purple corn supplements), light colored-bars represent the head withdrawal threshold evaluated at 48 h post CFA injection in the morning, whereas the dark colored-bars represent the head withdrawal threshold evaluated in the afternoon, 30 min after i.p. saline or 50 mg/kg ASA. Similar results have been obtained at 24 and 72 h post CFA injection (not shown). *N* = 8 (Water) and 9 (Yellow and Red) rats treated with saline, and 8 (Water) and 11 (Yellow and Red) rats treated with ASA. **p < 0.01 with respect to Red extract, two-way ANOVA followed by Bonferroni test; ^#^*p* < 0.05 with respect to corresponding value in the morning, Students’ *t*-test.

The average volume of water drunk by each animal was evaluated during a 3-day period. Identical quantities of Yellow and Red extracts were then dissolved in water, so that a daily amount of 53 mg of ACNs/kg body weight was administered to animals. Control animals received water. Administration started at Day-11 with respect to Complete Freund’s Adjuvant (CFA) injection, i.e., when the training was started, and was continued up to animals’ sacrifice (see Figure 1A).

### Induction of TMJ Inflammation and Administration of ASA

Rats were anesthetized with 1.5% isoflurane and unilateral subchronic TMJ inflammation was induced by the injection of 60 μl of CFA (Sigma-Aldrich, Milan, Italy) oil/saline 1:1 emulsion into the left TMJ capsule, as described (Villa et al., 2010; Magni et al., 2015). The site of injection was identified by palpating the zygomatic arch and condyle, and the injection was delivered by advancing a 27-gauge needle medioanteriorly through the skin immediately below the posteroinferior border of the zygomatic arch until it entered into the joint capsule. Injection was slowly performed over 1 min. In selected experiments, some CFA-injected rats drinking water or Yellow/Red extracts received 50 mg/kg ASA in 2 ml saline solution i.p. at 24, 48 and 72 h post CFA injection (in the afternoon; Magni et al., 2015). Saline was administered as control to a different group of CFA-injected rats drinking water or Yellow/Red extracts (see Figure 1A).

### Evaluation of Orofacial Allodynia by Von Frey Behavioral Test

Orofacial mechanical allodynia was measured as described (Villa et al., 2010; Magni et al., 2015). Briefly, a 1-week training was performed before CFA injection to get the animals acquainted with the operator and with the procedure. Diurnal time variations were carefully avoided. Unrestrained rats were probed with Von Frey filaments on the left and right orofacial skin region, near to the center of the vibrissae pad. Filaments were utilized in ascending series, with the starting filament corresponding to Log unit 4.31 (force: 2g). Each filament was tested five times at an interval of few seconds, and the response threshold was defined as the lowest force eliciting at least three head withdrawal responses out of five tests. The elapsed time between different filaments was 2 min. Thus, a specific pre-CFA injection head withdrawal threshold value was calculated for each animal. After CFA injection, evaluation of mechanical allodynia was performed 24, 48 and 72 h later (in the morning; Figure 1A). For sensitized inflamed animals, the force of the starting filament was reduced to Log unit 3.22 (force 0.16 g). In selected experiments, ASA or saline were administered 24, 48 and 72 h post CFA injection (in the afternoon; see above), and the Von Frey test was repeated 30 min later (Figure 1A).

### Immunohistochemistry

Rats were deeply anesthetized with ketamine (90 mg/kg) and xylazine (10 mg/kg), and transcardially perfused with PBS followed by 4% formalin, as previously described (Villa et al., 2010). The left and right (ipsi- and contra-lateral to the side of CFA injection, respectively) trigeminal ganglia (TG) and the brainstem were excised, postfixed in 4% formalin for 60–90 min, cryoprotected in 30% sucrose (48 h), embedded in mounting medium (OCT; Tissue Tek, Sakura Finetek, Zoeterwoude, Netherlands), and cut longitudinally on a cryostat at 15 μm thickness. TGs from each animal were embedded together.

TG and brainstem (at the level of the medulla oblongata; Villa et al., 2010) sections were then immunostained with a rabbit primary antibody against the microglia marker ionized calcium binding adaptor molecule 1 (anti-Iba1, 1:500; Wako, Richmond, VA, USA) followed by a goat anti-rabbit secondary antibody conjugated to AlexaFluor^®^488 (1:600; Thermo Fisher Scientific, Monza, Italy). The specificity of the staining was evaluated by incubating sections with secondary antibody only (Supplementary Figure S1). Cell nuclei were counterstained with the Hoechst33258 dye (1:20,000; Sigma-Aldrich, Milan, Italy).

#### Image Analysis

##### Cell Counting

The number of Iba1+ cells was counted in whole TG or brainstem slices acquired at 20× magnification and expressed as number of cells/area in μm^2^. At least three samples from three animals were evaluated.

##### Evaluation of Microglia Morphology and Branch Complexity

Digital images of whole Iba1-immunostained brainstem sections were acquired at 20× magnification and converted to binary grayscale to better analyze microglial cells morphology. The average cell size and the integrated optical density were evaluated by the “Particle analysis” tool of the Fiji-ImageJ software (Schindelin et al., 2012).

### Primary Microglia Cultures and *in vitro* Pharmacological Treatments With Corn Extracts

Primary microglia cultures were prepared as described (Chen et al., 2007). Briefly, mixed glia cultures were obtained from P2 Sprague-Dawley rat cortex and plated in 75-cm^2^ flasks in DMEM high glucose supplemented with 20% FBS. After 7 days in culture, microglia were detached from the underlying cells by shaking the flasks for 20 min at 200 rpm and plated into poly-D-lysine coated plates in DMEM high glucose supplemented with 15% FBS. Cells were seeded at 0.7–1 × 10^6^ cell/well in 12-well plates coated with poly-D-lysine (0.1 mg/ml) and overnight treated with Yellow or Red extracts (at a concentration corresponding to 125 μM ACNs, considering cyanidin 3-glucoside (C3G) MW: 484.84 g/mol, as the most abundant component) or medium alone, followed by a 6-h challenge with 1 μg/ml LPS (or medium as control). At the end of incubation, the medium was discarded, adherent cells were rinsed twice with ice-cold PBS, and lysed with 300 μl/well Qiazol^®^ Lysis Reagent (Qiagen, Hilden, Germany) for RNA extraction.

### Real Time RT-PCR Assays

Total RNA was isolated from cells with Direct-Zol™ RNA MiniPrep kit (Zymo Research, Irvine, CA, USA). About 1 μg RNA was reverse-transcribed with RT Superscript™ II (Invitrogen, Carlsbad, CA, USA) and Real time RT-PCR performed with Fast Evagreen qPCR Master Mix (Bio-Rad, Segrate, Italy) in a CFX96 real-time PCR detection system (Bio-Rad). The expression of each transcript was normalized against GAPDH and values were expressed as Fold Change over control sample (CNT). Primer sequences are reported in Table 1. Data are presented as mean ± SEM.

**Table 1:** Primer sequences utilized for real time RT-PCR.

Gene	Primer sequence 5′-3′
TNF-α-fw	GCAGATGGGCTGTACCTTATC
TNF-α-rv	GAAATGGCAAATCGGCTGAC
IL-6-fw	GCCAGAGTCATTCAGAGCAATA
IL-6-rv	TTAGGAGAGCATTGGAAGTTGG
IL-10-fw	AGTGGAGCAGGTGAAGAATG
IL-10-rv	GAGTGTCACGTAGGCTTCTATG
iNOS-fw	GTGGCTGTGGTCACCTATC
iNOS-rv	GTCTTCGGGCTTCAGGTTATT
MCP1-fw	CTGGCAAGATGATCCCAATGA
MCP1-rv	TCTCTTGAGCTTGGTGACAAATA
Arg1-fw	ACAAGACAGGGCTACTTTCAGG
Arg1-rv	ACAAGACAAGGTCAACGCCA
IL1β-fw	TGGCAACTGTCCCTGAACTC
IL1β-rv	GTCGAGATGCTGCTGTGAGA
YM-1-fw	GACACAATCCAGTCTGGTTACA
YM-1-rv	CAGTGTCGGATGGAGATTGATAG
Fizz1-fw	GAAGACCCTCTCATGCACTAAT
Fizz1-rv	ACAGGCAAAGCCACAAGA
IL-13-fw	ACATCACACAAGACCAGAAGAC
IL-13-rv	GGAGATGTTGGTCAGGGATTC
GAPDH-fw	TGTGAACGGATTTGGCCGTA
GAPDH-rv	ATGAAGGGGTCGTTGATGGC

*fw, forward; rv, reverse*.

### Western Blotting

Primary microglia were lysed in a buffer containing 50 mM Tris–HCl, pH 7.2, 0.1% sodium deoxycholate, 1% Triton X-100, 5 mM EDTA, 5 mM EGTA, 150 mM NaCl, 40 mM NaF, 2.175 mM NaVO_4_, 0.1% SDS, 0.1% aprotinin and 1 mM PMSF. Ten micrograms of protein underwent SDS–PAGE following transfer on nitrocellulose membranes. Bands were detected using Pierce™ ECL Western Blotting Substrate (ThermoFisher Scientific, Waltham, MA, USA) on a Chemidoc digital imaging machine. We utilized an anti-iNOS rabbit primary antibody (1:200 in 1% BSA; Cayman Chemical, Ann Arbor, MI, USA) followed by a goat anti-rabbit horseradish peroxidase-conjugated secondary antibody (1:3,000; Bio-Rad, Hercules, CA, USA). Internal loading control was performed by anti-α-Tubulin mouse primary antibody (1:500 in 1% BSA; Sigma-Adrich, St. Louis, MO, USA) followed by a goat anti-mouse horseradish peroxidase-conjugated secondary antibody (1:1,000; Sigma-Aldrich, St. Louis, MO, USA; see Supplementary Figure S2 for antibody specificity). Densitometry quantification was performed with ImageJ Software (NIH) and expressed as ratio of iNOS to α-tubulin.

### Statistics

Groups were compared by one- or two-way ANOVA followed by Bonferroni or Tukey *post hoc* tests for multiple comparisons or by Student’s *t*-test, when appropriate, as reported in the Legends to Figures using GraphPad Prism 6.0 software. *p* < 0.05 was considered statistically significant.

## Results

### Anti-allodynic Effect of Red Extract in a Rat Model of Trigeminal Sensitization and Comparison With Acetyl Salicylic Acid (ASA)

We first evaluated the effect of a prolonged (i.e., up to 14 days) consumption of purple corn supplement (Red extract) on facial mechanical allodynia due to CFA-induced TMJ inflammation (see Figure 1A for a schematic representation of the experimental protocol). Starting 24 h post CFA injection, rats drinking water showed a significant reduction in the head withdrawal threshold at Von Frey test with respect to the mean value pre-CFA injection (Figure 1B; from 30.81 ± 2.21 of “pre” value to 8.76 ± 0.62 at 24 h post CFA), which lasted until animals’ sacrifice (i.e., 8.56 ± 0.92 at 72 h post CFA-injection). Rats drinking yellow corn supplement (Yellow extract) showed an identical pattern of response, whereas after administration of Red extract a significantly higher value of the head withdrawal threshold (19.82 ± 2.07 at 24 h) was observed, and stably lasted up to sacrifice, indicating a partial but significant prevention of the development of mechanical allodynia by Red extract.

We next asked whether Red extract can synergize with a known anti-inflammatory drug, i.e., ASA, that we already demonstrated to exert significant anti-allodynic action on CFA-injected animals (Magni et al., 2015). When comparing the values of the head withdrawal threshold measured 48 h post CFA injection (light-colored bars in Figure 1C) or 30 min after ASA administration in the afternoon of the same day (dark-colored bars in Figure 1C), a marked anti-allodynic action of ASA was unveiled in the Water and Yellow groups, while a much smaller improvement was observed in the Red group. This is a consequence of the significant anti-allodynic effect exerted by the Red extract alone (Figure 1C, light red bars), which rose the head withdrawal threshold to a mean value comparable to values observed after ASA administration in the other two groups (Figure 1C, histograms on the right). Thus, we can conclude that a prolonged ingestion of Red extract is highly effective in preventing the development of allodynia in animals exposed to inflammatory trigeminal sensitization.

### Red Extract and ASA Are Equally Effective in Reducing CFA-Induced Trigeminal Infiltration of Macrophages

**Figure 2:**
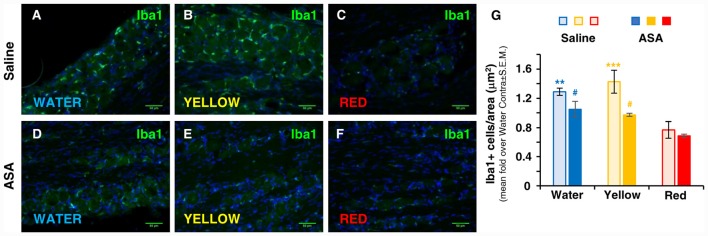
Red extract is as effective as ASA in inhibiting the trigeminal infiltration of Iba1+ macrophages in CFA-injected rats. **(A–F)** Representative micrographs of Iba1 staining (green) in the ipsilateral trigeminal ganglia (TG) of CFA-injected animals drinking water **(A,D)**, Yellow **(B,E)** or Red **(C,F)** extracts, treated with saline solution **(A–C)** or 50 mg/kg ASA **(D–F)**. Nuclei have been counterstained with the Hoechst33258 dye. Scale bars: 50 μm. The specificity of the anti-Iba1 primary antibody is shown in Supplementary Figure S1. **(G)** Evaluation of the number of Iba1+ cells/area in the ipsilateral TG of CFA-injected animals drinking water, Yellow or Red extracts and treated with saline (light colored-bars) or 50 mg/kg ASA (dark colored-bars). Animals have been sacrificed at 72 h post CFA injection (see protocol in Figure 1 and text for details). *N* = 4 (Water), 3 (Yellow) and 5 (Red) rats treated with saline, and three (Water, Yellow and Red) rats treated with ASA. **p < 0.01 and ***p < 0.001 with respect to purple corn supplement, two-way ANOVA followed by Bonferroni test; ^#^*p* < 0.05 with respect to corresponding Saline values, Students’ *t*-test.

The contribution of inflammatory cells, which migrate from the bloodstream and infiltrate the trigeminal ganglion, to the development of trigeminal-related painful conditions has been clearly established (Villa et al., 2010; Franceschini et al., 2013; Batbold et al., 2017; Wang et al., 2018). We therefore evaluated the number of trigeminal macrophages, identified by the expression of Iba1, under the various experimental conditions 72 h after CFA injection. In parallel with the effect on the development of mechanical allodynia (see Figure 1), a significantly lower density of Iba1+ cells in the trigeminal ganglion with respect to water and Yellow extract was observed in animals drinking Red extract (see representative pictures in Figures 2A–C and quantification of data in histograms in Figure 2G). Additionally, administration of ASA to animals receiving either Water or Yellow extract was as effective as Red extract alone in reducing macrophage infiltration (Figures 2D–F), but ASA showed no additional effect on the Red extract group (dark columns in Figure 2G). These data suggest that Red extract-induced prevention of orofacial allodynia proceeds through a peripheral anti-inflammatory effect, which resembles the well-known effect of a marketed NSAID.

### Exclusive Effect of Red Extract on Microglia Activation *in vivo* and *in vitro*

**Figure 3:**
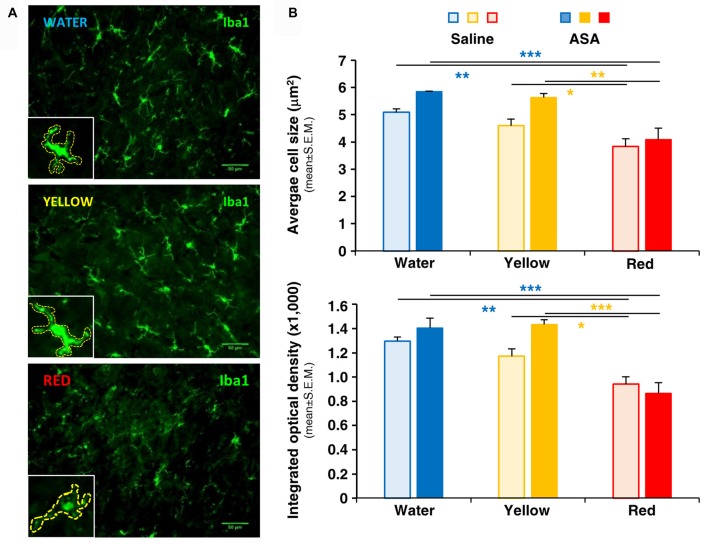
Red extract inhibits the activation of Iba1+ microglia in the brainstem of CFA-injected rats. No effects of ASA. **(A)** Representative pictures of Iba1+ microglia in the medulla oblongata of the brainstem of CFA-injected rats drinking water, yellow or purple corn extracts (from top to bottom). Scale bars: 50 μm. Insets: magnification of representative microglia cells, showing thinner and feebly stained processes in rats drinking Red extracts with respect to rats drinking water or Yellow extract. Cells have been highlighted by dashed lines to perform quantitative analysis (see “Materials and Methods” section for details). **(B)** Histograms showing the average cell size (top) and the mean integrated optical density of Iba1 staining (bottom) in CFA-injected animals drinking water, Yellow or Red extracts and treated with saline (light colored-bars) or 50 mg/kg ASA (dark colored-bars). Animals have been sacrificed at 72 h post CFA injection (see protocol in Figure 1 and text for details). *N* = 4 (Water), 3 (Yellow) and 5 (Red) rats treated with saline, and three (Water, Yellow and Red) rats treated with ASA. *p < 0.05, **p < 0.01 and ***p < 0.001 with respect to corresponding purple corn supplement, two-way ANOVA followed by Bonferroni test.

At central level, the development of trigeminal pain is directly linked to the activation of microglia in the medulla oblongata in the brainstem and in the cervical spinal cord (Villa et al., 2010; Wang et al., 2018), i.e., in CNS areas in connection with the trigeminal nerve. We have therefore deemed interesting to analyze the possible effect of Red extract on microglia cell number and shape changes, a typical correlate of their transition from a resting to a pro-inflammatory phenotype (Walker et al., 2014; Crews and Vetreno, 2016). Immunohistochemical analysis of Iba1 staining in the brainstem did not reveal differences in the total number of positive cells among the three experimental groups (number of Iba1+ cells/area in μm^2^: 0.179 ± 0.020, 0.173 ± 0.022 and 0.166 ± 0.028 in the Water, Yellow and Red group, respectively; not statistically significant, five slices from two animals/condition). Conversely, striking differences in microglia cell morphology were observed between the Red and the Water/Yellow groups, as shown by insets in representative pictures in Figure 3A. Thicker and highly stained cell processes, indicative of microglia activation, were in fact detected in both the Water and Yellow groups. Administration of Red extract significantly reduced both cell size and Iba1 staining intensity (see quantification in Figure 3B), with microglia morphology characterized by very thin processes, typical of a resting phenotype (see Figure 3A, inset). Concomitant administration of ASA did not modify either parameter of microglia activation in any experimental condition (Figure 3B). This suggests multiple mechanisms of action for Red extract, targeting both infiltrating cells in peripheral tissues (see above) and resident glia cells in the CNS, whereas ASA exerts only a peripheral anti-inflammatory action.

**Figure 4:**
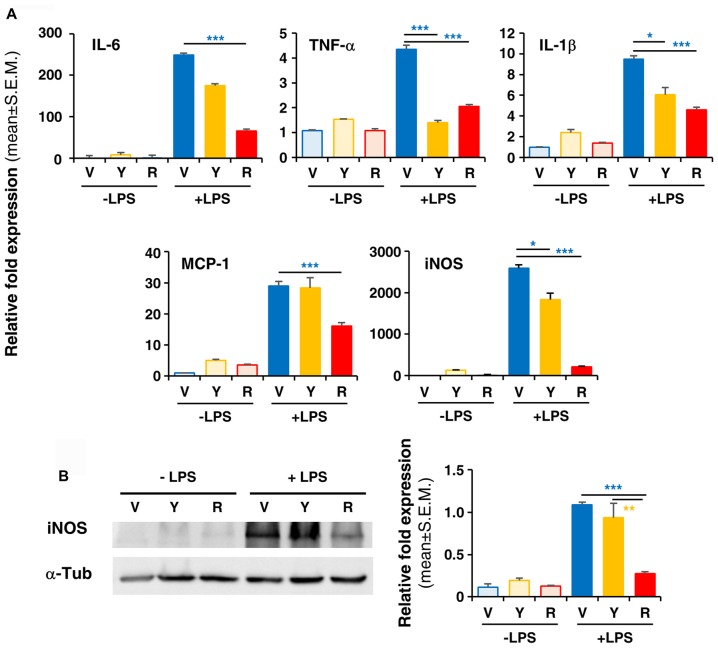
*In vitro* treatment of LPS-activated rodent microglia with Red extract reduces the production of pro-inflammatory mediators. Rodent microglia cells were grown in culture, overnight exposed to medium alone, Yellow or Red extracts, and then challenged with medium alone or 1 μg/ml LPS for 6 h (see “Materials and Methods” section for details). Cells were then collected and the expression of pro-inflammatory cytokine genes was evaluated by Real time RT-PCR **(A)**. Each transcript was normalized to corresponding control value (V, vehicle). Values shown are mean ± SEM from two independent experiments performed in triplicate. **(B)** Evaluation of iNOS protein levels by Western blotting and relative quantification. Filter is from one representative experiment out of three (see Supplementary Figure S2 for the specificity of antibodies). Densitometric analysis of bands have been performed with ImageJ and values normalized to α-tubulin. Histograms show the mean ± SEM values from three independent experiments performed in triplicate. *p < 0.05, **p < 0.01, and ***p < 0.001 with respect to cultures exposed to Red extract, one-way ANOVA followed by Bonferroni *post hoc* tests.

**Figure 5:**
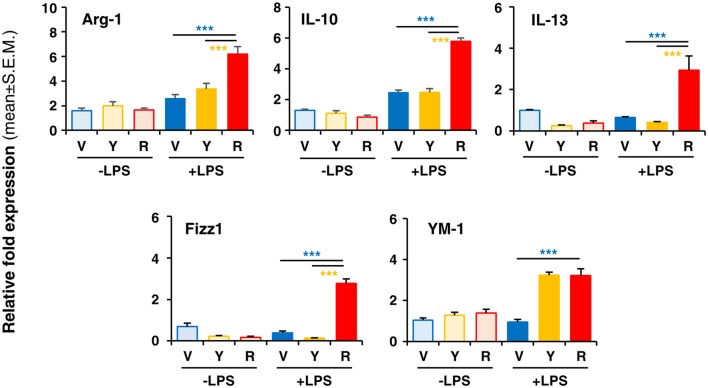
*In vitro* treatment of LPS-activated rodent microglia with Red extract induces the expression of anti-inflammatory mediators. Rodent microglia cells were grown in culture, overnight exposed to medium alone, Yellow or Red extracts, and then challenged with medium alone or 1 μg/ml LPS for 6 h (see “Materials and Methods” section for details). Cells were then collected, and the expression of anti-inflammatory genes was evaluated by Real time RT-PCR. Each transcript was normalized to corresponding control value (V, vehicle). Values shown are mean ± SEM from two independent experiments performed in triplicate. ***p < 0.001 with respect to cultures exposed to Red extract, one-way ANOVA followed by Bonferroni *post hoc* tests.

To verify whether Red extract can directly influence microglia functions, we have pre-treated primary microglia cultures with medium alone, Yellow or Red extracts and then simulated cell activation by incubation with LPS. The expression of pro- or anti-inflammatory mediators has been then evaluated by Real Time RT-PCR. As shown in Figure 4A, pre-treatment of cultures with Red extract completely reverted LPS-promoted expression of pro-inflammatory IL-6, TNF-α, IL-1β, MCP-1 and iNOS. Additionally, we verified whether the reduced transcript level of the iNOS gene correlated with a decrease in iNOS protein. The expression of iNOS protein was up-regulated in LPS-induced primary microglia cells, but significantly inhibited to the level of the untreated control group when cells were pre-treated with Red extracts (Figure 4B). At the same time, Red extract induced a significant up-regulation of anti-inflammatory mediators, such as Arg-1, IL-10, Fizz1 and IL-13 and of the marker of anti-inflammatory microglia phenotype YM-1 (Figure 5). Yellow extract was partially effective in reducing the expression of some of the pro-inflammatory mediators and only upregulated YM-1 among anti-inflammatory genes (Figures 4A, 5). Taken together, these data suggest a direct action of Red extract on microglia function and activation.

## Discussion

### Non-neuronal Cells as Targets for the Management of Trigeminal Allodynia

The present work is the first demonstration of the anti-allodynic effect of purple corn extract in a rat model of trigeminal sensitization, which involves both peripheral infiltrating macrophages and central glial cells. Targeting non-neuronal cells, including macrophages and glial cells, has now emerged as a promising and effective approach for many painful conditions, including head and face pain and allodynia. For example, both peripheral macrophages and central microglia are involved in the development of chronic pain due to forced prolonged mouth opening and TMJ inflammation, and inhibiting their activation leads to significant amelioration of pain (Wang et al., 2018). Additionally, macrophages infiltrating the trigeminal ganglion are directly involved in the development of ectopic orofacial pain following the transection of the inferior alveolar nerve, and their depletion or the blockade of inflammatory pathways are protective against allodynia (Batbold et al., 2017). We previously demonstrated that macrophage trigeminal infiltration and central microgliosis are hallmarks of inflammatory trigeminal pain due to CFA injection in the TMJ (Villa et al., 2010), the animal model utilized in the present study, suggesting that these cell types significantly contribute to the development of pain.

### The Anti-inflammatory Effects of Anthocyanins Involve the Shift of Microglia Towards a Neuroprotective Phenotype

The Janus face of microglia cells is demonstrated by their ability to contribute and sustain inflammation or to promote brain tissue repair and resolution of inflammatory processes, thanks to the expression of either pro- or anti-inflammatory mediators, respectively. This has led to the long-utilized sub-classification of microglia cells as M1 (detrimental) or M2 (reparative) phenotypes (Kierdorf and Prinz, 2013; Orihuela et al., 2016). Although this classification is currently debated by several authors, as too simplistic to adequately mirror the vast variety of intermediate activation states of these cells (Ransohoff, 2016), it is still undoubted that microglia cells can be activated and respond to changes in their extracellular milieu by playing either detrimental or beneficial roles. It has been demonstrated that ACN treatment inhibits LPS-induced microglial activation *in vitro* (Poulose et al., 2012; Jeong et al., 2013). However, despite increasing fractalkine expression, single purified ACNs (i.e., cyanidin, C3G, or methylated C3G) did not significantly promote microglial polarization towards an anti-inflammatory phenotype (Meireles et al., 2016). ACNs can cross the blood-brain barrier in significant amounts following systemic administration (Hribar and Ulrih, 2014), and therefore a protective effect can be predicted also *in vivo*. A very recent article demonstrated the beneficial effects of ACNs on the CNS in a mouse model of LPS-induced neuroinflammation and memory loss, with a significant reduction of microglia activation after administration by oral gavage of ACNs purified from bilberry (Carvalho et al., 2017). Our results confirm and extend these published data, demonstrating that purple corn extract inhibits microglia activation and promotes their shift towards the production of anti-inflammatory mediators and that more than one component within purple corn ACNs may promote this shift. Furthermore, we have utilized a crude purple corn extract which can be easily prepared at a lower cost than purified ACNs. Based on the amount of Red supplement drunk by animals, we calculated that about 53 mg ACNs/kg body weight have been assumed daily by animals, in line with the amount of ACNs administered by gavage in published studies (Carvalho et al., 2017). Being animals free to drink the Red extract dissolved in water, instead of being forcedly administered to oral gavage, represents an important add-on of our protocol which demonstrates animals’ compliance to the treatment and more closely resembles a possible clinical administration.

Concerning the possible molecular mechanisms at the basis of the anti-inflammatory effects of Red extract, our data show a significant reduction of iNOS expression *in vitro*. This could be related to the activation of PPAR-γ receptors which have already been demonstrated to inhibit LPS-stimulated iNOS expression and TNF-α production as well as IFN-γ-induced expression of major histocompatibility complex class II antigens, by preventing the activation of the transcription factors STAT-1 and NF-κB in rodent microglia *in vitro* (Bernardo and Minghetti, 2006). ACNs from purple corn have been recently demonstrated to activate PPAR-γ receptors in adipocytes (Luna-Vital et al., 2017), thus it will be worth evaluating whether this signaling pathway can be activated in microglia cells as well. Additionally, it has been shown that ACNs exert their anti-inflammatory properties by inhibiting the LPS- or cytokine-induced nuclear translocation of the transcription factor NF-kB in multiple cell types (Kim et al., 2006; Limtrakul et al., 2015; Ferrari et al., 2016). Thus, a possible inhibition of NF-kB-mediated signaling pathways by purple corn ACNs can also be speculated in microglia cells.

### Other Possible Mechanisms Implicated in the Anti-allodynic Action of Red Extract in Trigeminal Pain

Besides its direct action on microglia cells, we cannot exclude that other more indirect mechanisms are involved in the observed effect of Red extract. Thanks to its oral prolonged administration, we can foresee its possible influence on the composition of gut microbiota. A wealth of studies has been recently published demonstrating a close interconnection between commensal bacteria in the intestine and brain development and functions (reviewed in Sharon et al., 2016; Tognini, 2017; Gilbert et al., 2018), so that alterations in microbiota composition is now considered to be at the basis of many neurological disorders. It is now emerging that ACNs are metabolized by intestinal microbiota, and, on the other hand, they and/or their metabolites can modulate the growth of specific bacterial taxa (Faria et al., 2014). A direct effect of the so-called gut-brain axis on microglia functions has been highlighted (Chunchai et al., 2018), but no data are currently available to correlate gut microbiota, microglial cells and the development of orofacial pain. In future studies, we aim at verifying the involvement of this important correlation in our experimental model.

### Future Clinical Exploitation of the Anti-allodynic Effects of Red Extract as Adjuvant in Pain Management

Whatever the molecular mechanism(s) involved, the peculiar action of Red extract which exerts beneficial effects both in the periphery and at the central level represents an innovative preventive approach for the development of trigeminal-related allodynia as a consequence of inflammatory pain. In fact, inhibiting the recruitment to the pain pathways and the activation of central glial cells could help controlling and managing not only acute painful behaviors but, more importantly, their transition to persistent chronic painful conditions, which are triggered and sustained by glia-mediated maladaptive plasticity of central brain circuits (Ji et al., 2013, 2016; Grace et al., 2014). Thus, the prolonged administration of Red extract could prove beneficial in limiting pain responses to acute conditions that will not eventually develop to chronic situations. In fact, although a significant reduction of allodynia can be observed in the presence of a peripheral site of action only, as demonstrated by our data on the effect of ASA on macrophages infiltrating the trigeminal ganglion, the additional ability of purple corn to also prevent the activation of central microglia represents a significant advantage.

Although significant concentrations of ASA have been detected in the CNS after systemic administration (Ajmone-Cat et al., 2010), we could not detect any influence on microglia activation in our experimental model. The most plausible hypothesis is that when administered after the induction of trigeminal sensitization, as in our experimental protocol, ASA cannot reach a sufficient brain concentration to revert microglia activation but can only modulate and reduce the peripheral events controlling orofacial allodynia. Due to its significant gastric side effects and to its negative modulation of blood clotting, a preventive and prolonged exposure to analgesic concentrations of ASA, and NSAIDs in general, is not recommended. On the other side, purple corn extract has already proved its safety in humans in one currently ongoing clinical trial on breast cancer patients undergoing radiotherapy (Cerletti et al., 2017). Thus, lacking any significant side effects, purple corn extract represents an innovative option for the prevention of orofacial pain following, for example, dental surgery.

ACNs in purple corn mostly consist of C3G and its malonyl derivatives, whereas peonidin 3-glucoside and pelargonidin 3-glucoside are present only in small amounts (Petroni et al., 2014, 2017). Since all other phenolic acids and flavonoids are identical between purple and yellow corn (Pilu et al., 2011), the anti-allodynic effect of purple corn extract may be mainly attributed to C3G and its derivatives. Nonetheless, animals drank similar amounts of water and of the two corn extracts, thus allowing us to exclude any influence of the quantity of swallowed supplement on the observed behavioral effects.

The anti-inflammatory effects of ACNs from purple corn have been previously demonstrated in several models of systemic inflammation, in particular liver inflammation linked to diabetes-like conditions (Li et al., 2012; Bhaswant et al., 2017). ACNs from Korean black soybean have been proved efficacious in reducing neuroinflammation in a rat model of Alzheimer’s disease (González-Reyes et al., 2017). No data are currently available on their role in inflammatory painful conditions, and in modulating glial cell reactivity. Thus, our data support for the first time the use of ACN-based supplements or functional foods as adjuvant therapy for chronic pain conditions, targeting glial cells (Möller and Boddeke, 2016).

Taken together, our results demonstrate a significant prevention of the development of orofacial allodynia induced by trigeminal inflammation by purple corn extract and suggest that it represents a promising supplement for the control of trigeminal pain, accompanied by a cost-efficient production and no side effects.

## Author Contributions

GM performed *in vivo* studies and immunohistochemical analyses and analyzed data. AM performed *in vitro* experiments, qRT-PCR analyses and analyzed data. DR collaborated to *in vivo* studies. DL prepared microglia primary cultures. CT and MA discussed data and contributed scientific advices. KP coordinated *in vitro* experiments, analyzed and discussed data and provided purple and yellow corn extracts. SC coordinated *in vivo* studies, analyzed and discussed results and drafted the manuscript. All authors contributed corrections and ideas for the manuscript and approved it in its final form.

## Conflict of Interest Statement

The authors declare that the research was conducted in the absence of any commercial or financial relationships that could be construed as a potential conflict of interest. The reviewer SV and the handling Editor declared their shared affiliation.
