# Supplementary material for: Purple Corn Extract as Anti-allodynic Treatment for Trigeminal Pain: Role of Microglia
Source: Front Cell Neurosci. 2018 Nov 5;12:378. doi: 10.3389/fncel.2018.00378 (PMC6230559; doi:10.3389/fncel.2018.00378)
Supplement: Supplementary file 1 [file Data_Sheet_1.PDF]

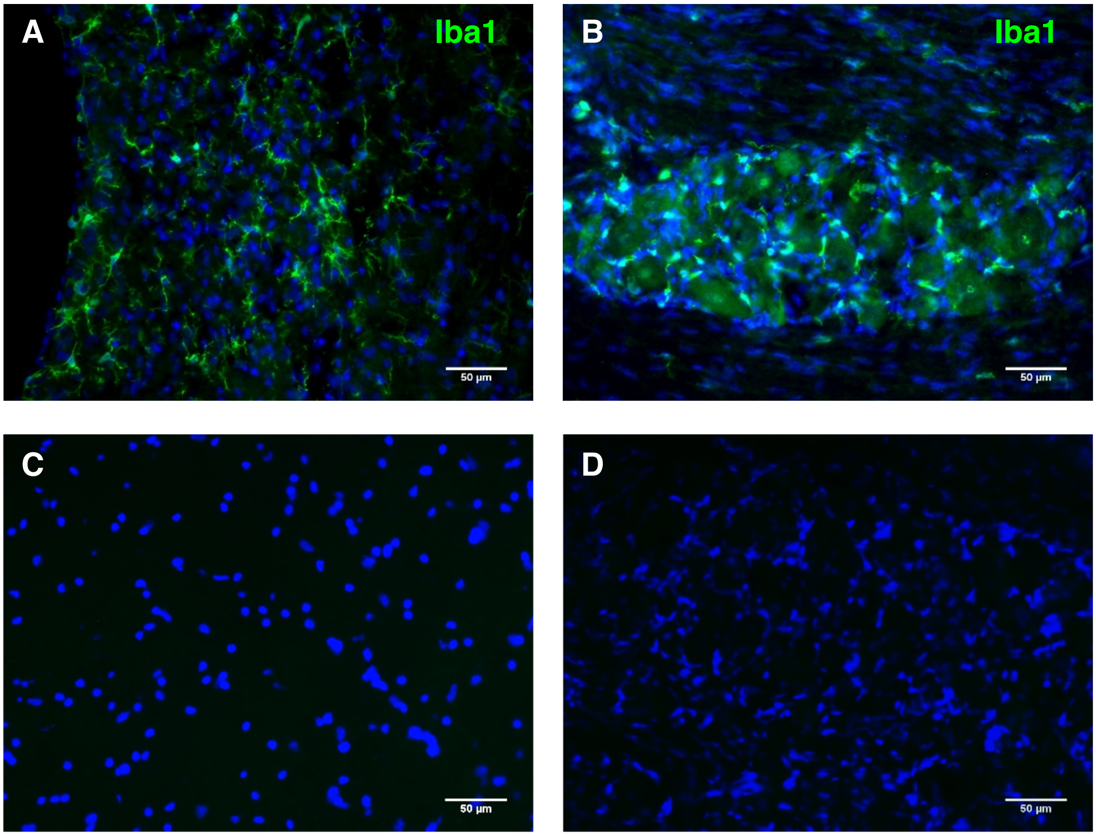

Supplementary Figure 1. Specificity of Iba1 immunostaining.

(A, B) Iba1 immunofluorescence staining (green) in the brainstem (A) and TG (B) of CFA-injected rats. **(C, D)** The specificity of anti-Iba1 primary antibody was determined by incubating slices from brainstem (C) and TG (D) of CFA-injected rats with secondary antibody only. Nuclei were labeled with the Hoechst33258 dye (blue). Scale bars: 50 μM.
