# Supplementary material for: Purple Corn Extract as Anti-allodynic Treatment for Trigeminal Pain: Role of Microglia
Source: Front Cell Neurosci. 2018 Nov 5;12:378. doi: 10.3389/fncel.2018.00378 (PMC6230559; doi:10.3389/fncel.2018.00378)
Supplement: Supplementary file 2 [file Data_Sheet_2.PDF]

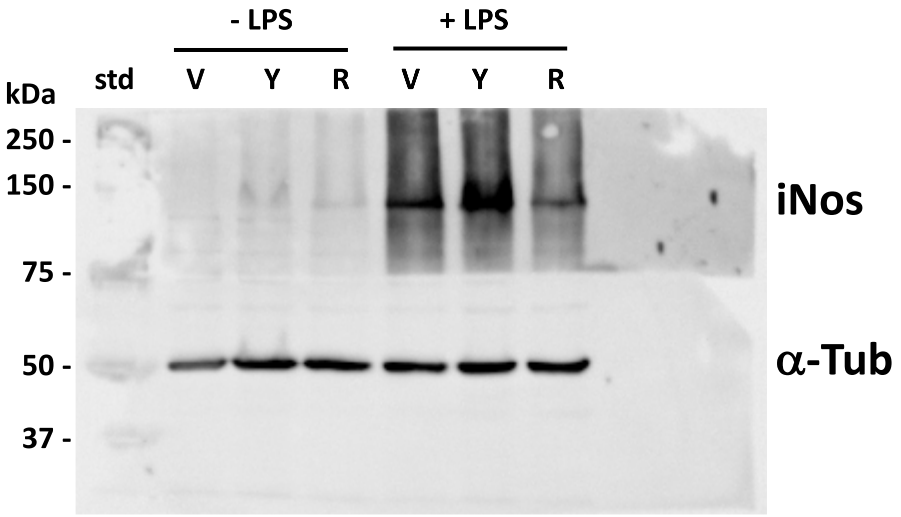

Supplementary Figure 2. Specificity of anti-iNos and anti-α-Tub primary antibodies for Western blotting.

After protein transfer, nitrocellulose filter has been cut in two parts which have been incubated with anti-iNos (upper part) or anti-α-Tub primary antibodies, followed by species-specific horseradish peroxidase-conjugated secondary antibodies and ECL detection (see Methods). The image of the original filter shows that only specific bands at the expected molecular weights (i.e., 131 kDa and 50 kDa for iNOS and α-Tub, respectively) are detected. std: molecular weight markers (Bio-Rad #161-0374)
